# Practitioner review: Co-design of digital mental health technologies with children and young people

**DOI:** 10.1111/jcpp.13258

**Published:** 2020-06-22

**Authors:** Rhys Bevan Jones, Paul Stallard, Sharifah Shameem Agha, Simon Rice, Aliza Werner-Seidler, Karolina Stasiak, Jason Kahn, Sharon A. Simpson, Mario Alvarez-Jimenez, Frances Rice, Rhiannon Evans, Sally Merry

**Affiliations:** 1 Division of Psychological Medicine & Clinical Neurosciences, Cardiff University, Cardiff, UK; 2 National Centre for Mental Health, Cardiff University, Cardiff, UK; 3 Cwm Taf Morgannwg UHB, Wales, UK; 4 Department for Health, University of Bath, Bath, UK; 5 Orygen, Parkville, VIC, Australia; 6 Centre for Youth Mental Health, University of Melbourne, VIC, Australia; 7 Black Dog Institute, UNSW Sydney, Sydney, NSW, Australia; 8 Faculty of Medical and Health Sciences, University of Auckland, Auckland, New Zealand; 9 Boston Children’s Hospital, Harvard Medical School, Boston, MA, USA; 10 MRC/CSO Social and Public Health Sciences Unit, Institute of Health and Wellbeing, University of Glasgow, Glasgow, UK; 11 DECIPHer, Cardiff University, Cardiff, UK

**Keywords:** Child, adolescent, mental health, digital, technologies, e-health, development, co-design

## Abstract

**Background:**

There is increasing interest in digital technologies to help improve children and young people’s mental health, and the evidence for the effectiveness for these approaches is rising. However, there is concern regarding levels of user engagement, uptake and adherence. Key guidance regarding digital health interventions stress the importance of early user input in the development, evaluation and implementation of technologies to help ensure they are engaging, feasible, acceptable and potentially effective. Co-design is a process of active involvement of stakeholders, requiring a change from the traditional approaches to intervention development. However, there is a lack of literature to inform the co-design of digital technologies to help child and adolescent mental health.

**Methods:**

We reviewed the literature and practice in the co-design of digital mental health technologies with children and young people. We searched Medline, PsycInfo and Web of Science databases, guidelines, reviews and reference lists, contacted key authors for relevant studies, and extracted key themes on aspects of co-design relevant to practice. We supplemented this with case studies and methods reported by researchers working in the field.

**Results:**

We identified 25 original articles and 30 digital mental health technologies that were designed/developed with children and young people. The themes identified were as follows: principles of co-design (including potential stakeholders and stages of involvement), methods of involving and engaging the range of users, co-designing the prototype and the challenges of co-design.

**Conclusions:**

Co-design involves all relevant stakeholders throughout the life and research cycle of the programme. This review helps to inform practitioners and researchers interested in the development of digital health technologies for children and young people. Future work in this field will need to consider the changing face of technology, methods of engaging with the diversity in the user group, and the evaluation of the co-design process and its impact on the technology.

## Introduction

Mental health difficulties are common in children and young people (CYP), but most are not getting any help (Neufeld et al., 2017). Digital mental health technologies (i.e. resources and interventions to support and improve mental health) have been identified as a potential way to improve reach and access to therapies, at relatively low cost. There is growing evidence to support the use of some technologies (Hollis et al., 2017), with guidelines recommending, for example, digital cognitive behavioural therapy (CBT) for depression (National Institute for Health and Care Excellence., 2019). Furthermore, many CYP have access to the internet and mobile technologies, including in low and middle-income countries (LMIC; Naslund et al., 2017). However, a major challenge in this area is the low user engagement, uptake and adherence to these programmes outside research settings (Fleming et al., 2019; Hollis et al., 2017).

Over recent years, there has been an increase in the publication of general guidance for intervention development. Guidance specific to digital health (e.g. Mohr et al., 2014; Yardley et al., 2015) reflects a broader direction of travel in the development and evaluation of complex health interventions, which stress the importance of the development phase and of user input from the initial stages (Craig et al., 2008; Hawkins et al., 2017; Wight et al., 2016). Co-design is a process of ‘collective creativity’ or ‘partnership’ with potential users and stakeholders, who are actively involved across the entire development of the technology - helping to ensure it meets the user’s needs and preferences. A rigorous process involving users, relevant theory and research evidence is more likely to produce an intervention that is evidence-based, engaging, acceptable and feasible to deliver (Thabrew et al., 2018).

Whilst the principle of co-design of digital health technologies is becoming accepted, there is limited guidance and literature on how this can optimally be undertaken. Co-design requires a shift from the traditional practice of expert-led development work where interventions are designed ‘for’ to one where they are designed ‘with’ CYP (Hodson et al., 2019). Designing and producing with CYP helps to ‘humanise’ the field of digital technologies which some have criticised for being overly structured, rigid and unresponsive.

The development of technologies for child and adolescent mental health requires particular considerations, and they should not merely be adaptations from ‘adult programmes’. A developmental or age-appropriate approach is needed regarding the content and design of a programme, and accounting for the range of interests and tastes of CYP. The presentation and management of mental health difficulties in this age group are also different to that of adults (WHO, 1993).

We provide a practitioner review of the literature on the approaches to the design and development of digital mental health technologies in collaboration with CYP and other stakeholders. We will map the existing evidence and practice for the co-design with CYP and use case studies and exemplars to illustrate key points throughout. The review offers an overview of an emerging research area to practitioners and researchers and concludes with practice points to help guide the planning, reporting and analysis of co-design activities.

## Methods

Relevant articles were identified through computer searches in Medline, PsycInfo and Web of Science databases to July 2019, with no restriction regarding publication dates. The key search terms and methodology are outlined in Figure S1. Studies were appraised against the following inclusion criteria: articles with information on the (co-) design/development/production, of digital mental health technologies with and for CYP (up to 18 years); papers published or translated into English in a peer-reviewed journal. There was a focus on programmes/ applications to help with depression, anxiety, sleep, self-harm and suicide. We searched reviews, guidelines and reference lists, and contacted key authors with expertise in the development of digital mental health interventions for CYP, especially where it was unclear whether CYP were involved in the design/ development of certain technologies. Papers were excluded if technologies were developed for adults or primarily for physical health, or were diagnostic, screening, monitoring, communication or data management tools.

Titles and abstracts, and then full texts, were screened by RBJ and SSA. As this was an exploratory and descriptive review, an inclusive approach was taken to assessing full texts. Study quality was not appraised, as we aimed to map the emerging literature (rather than verifying an evidence base of effects) given the heterogeneous nature of the field. Data were extracted by RBJ and SSA on the co-design processes for a range of technologies and the findings were discussed with other authors. The data were categorised by the two authors into key overarching and recurring themes that help to understand the practical processes of involving CYP in the co-design of technologies. These themes were illustrated through case studies on specific technologies. The ‘Practice points’ section was based on the review findings and our methods, and reflections on future developments as researchers working in the field.

## Results

### Summary of studies

The original searches yielded 5891 articles after duplicates were removed, and 292 articleswere assessed from full text (see Figure S1). We identified 25 original articles and 30 digital mental health technologies that met the inclusion criteria (summarised in Table 1). Fifteen of the technologies (50%) were developed to help with several mental health difficulties, with eighteen of the technologies targeting depression (60%), nine targeting anxiety (30%), three targeting self-harm or suicidal ideation (10%), one targeting sleep (3%), and six helping with ‘general mental health’ difficulties or crises (20%). Twenty-nine technologies were developed with adolescents (97%), whilst five involved children (under 12 years; 17%).

Sixteen technologies were developed in Australasia (New Zealand, Australia; 53%), seven in North America (USA, Canada; 23%), five in Europe (England, Ireland, Wales; 17%) and two in Asia (Hong Kong; 7%). Nineteen articles (76%) focused primarily on the development phase of the technology (and the involvement of CYP in this), whilst six (24%) focused mainly on an evaluation or a trial of the technology (with briefer accounts of CYP involvement). Most articles that focused on the design/development were published in recent years (eleven since 2017).

The following key recurring themes related to the co-design process were identified: (a) the principles of co-design, including the participants/stakeholders and stages of involvement; (b) the potential methods and techniques of involving and engaging CYP; (c) co-designing the initial prototype, considering the diversity in the user group; and (d) the potential challenges of co-design with CYP, including its evaluation.

### Principles of co-design

#### Creative collaboration

Co-design originated in the field of participatory design, which emphasises the importance of involving all potential users and stakeholders as active collaborators in the development of a product. This aims to ensure that technologies meet the users’ range of needs and preferences and are acceptable and helpful. Studies described how the process can involve all aspects of the technology, including content, design, accessibility, usability, data management/security, integration and implementation into users’ lives and everyday context (e.g. Hodson et al., 2019; Robinson et al., 2017; Thabrew et al., 2018; Werner-Seidler et al., 2017; Wiljer et al., 2017). A central need for co-design in the context of complex interventions is to understand the interaction of the technology within the complex psychosocial system within which it attempts to enact change (Craig et al., 2008; Hawkins et al., 2017).

As well as CYP as the primary users of the technologies, studies described how co-design might involve the following, especially if they are potential users: (a) *families, carers and friends,* (b) *service practitioners/ experts* (e.g. in education, health, social, youth services) to explore issues such as facilitators and barriers to use, (c) *contentpractitioners/experts* (e.g. clinicians, researchers) to help determine the evidence-based content of the technology, (d) *practitioners with expertise in digital technologies*, including designers, information technology (IT) developers, animators, scriptwriters and model-makers.

The process goes beyond involving CYP as a consultation or engagement exercise, but embraces a ‘democratic partnership’ with appropriate distribution of power, jointly exploring needs and creating possible solutions with CYP as ‘experts of their experiences’ (Thabrew et al., 2018). To help achieve this, Hodson et al. (2019) describe four important elements: (a) engagement with users before the project starts; (b) acknowledging the potential power imbalance between practitioners/researchers and CYP, and involving all as both ‘providers’ and ‘recipients’; (c) establishing activities (e.g. workshops) where all participants collaborate to generate ideas, guided by a facilitator, to develop and refine the product, with users always ‘signing-off’ on design proposals; (d) practitioners/researchers creating the final product according to project requirements, with possible further collaboration with users. CYP might also gain knowledge, skills and career advice from the process (Oliver et al., 2006).

#### Stages of involvement

It has been advocated that co-design is a dynamic and continuous process, featuring throughout the life cycle of the technology (Craig et al., 2008; Hawkins et al., 2017). This review focuses mainly on co-design during the initial development of the technology, as most studies reported only on CYP involvement during this phase - ranging from involving a small number of users at one point, to more prolonged and in-depth collaboration. There can be certain prescribed stages of user involvement, and twelve articles described an initial ‘scoping’ or ‘discovery’ phase involving CYP and other stakeholders regarding their needs and preferences (and a review of the literature and practice), before establishing the focus of the process. In the development of new interventions, co-design can involve iterative design cycles and start with ‘superficial probes’ to engage users (involving mainly researchers and designers), followed by a more intense generation of ideas (mainly involving users), and finally a narrowing of focus on the development of a prototype (all collaborating equally; Thabrew et al., 2018). Fourteen articles also described an evaluation of the initial prototype.

As a case study of the iterative co-design process of a digital technology for depression, SPARX (Shepherd et al., 2015) followed the development of an early version of gamified CBT, The Journey (by the authors KS and SM). This started with a review of best practice, identification of key therapeutic elements and learning goals, and workshops with young people (YP) and learning-technology experts. A Flash-based program was developed, and a pilot trial showed this approach was effective, although feedback suggested that YP wanted several improvements. These findings informed the development of SPARX, which included YP, clinicians, computer games practitioners, cultural advisors (Māori, Pacific people, Asian) and researchers. After initial consultations, YP were not involved again until the development of the first SPARX prototype, and they were negative about its design. The subsequent format was designed by agroup of fourteen YP, with aprocess of lively discussion with the clinician researchers. The group was selected to be representative of YP in New Zealand in terms of age (13-17 years), ethnicity, socioeconomic group and gender, and recruited through schools, youth groups and personal contacts. The IT team and researchers then worked together weekly to implement the ideas of the YP, who were consulted throughout the process. At the end of development, YP were supportive of the approach taken and suggested further refinements.

**Figure 1:**
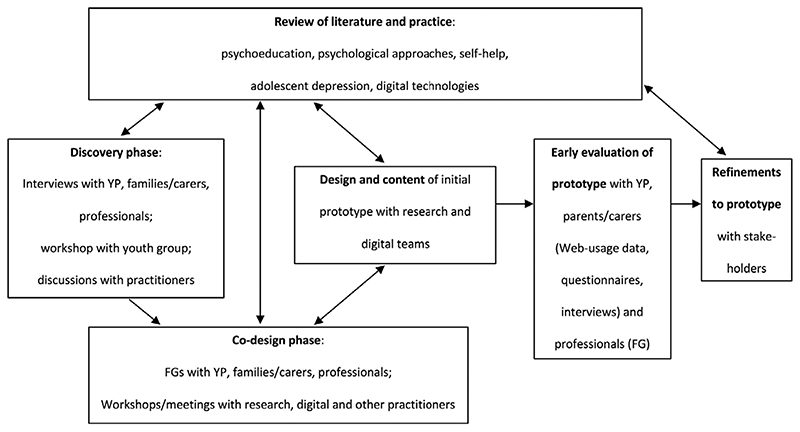
General framework for the development of the digital technology MoodHwb

As a second case study, Figure 1 presents the overall development of another technology for depression, MoodHwb (Bevan Jones, Thapar, Rice, et al., 2018). This broadly follows the frameworks described above, whereby the initial ideas for the project were generated mainly from interviews with YP (with depressive symptoms or ‘at-risk’ of depression, because of a family history), parents/carers and professionals from health, education, social and youth services (discovery phase), and a systematic review (Bevan Jones, Thapar, Stone, et al., 2018). This informed the subsequent codesign phase involving focus groups (FGs) and workshops with these groups, a digital team and experts in psychology/psychiatry, services and design. During the early evaluation, YP and parents/carers used the prototype. Web-usage, questionnaire and interview data were analysed to determine its initial feasibility and acceptability, and to inform its refinement (Bevan Jones et al., 2020).

### Methods of involvement and engagement

#### Collaborative activities

Children and YP with mental health difficulties might be reluctant to participate in research, because of the associated anxiety, embarrassment, stigma, motivational and other difficulties associated with mental health problems (Han et al., 2019; Hodson et al., 2019; Lucassen et al., 2013; Radovic et al., 2018; WHO, 1993). Techniques used in the studies to engage CYP included well-designed information sheets, consent forms, posters and websites outlining the aims and benefits of the study, and vouchers/expenses offered as a thank you (e.g. Bevan Jones, Thapar, Rice, et al., 2018). As with the SPARX study, other researchers attempted to recruit a diverse and representative range of participants, so as to capture the diversity in the user preferences. Eleven articles described recruiting from educational services (44%), 10 via community organisations and volunteers (40%), and five from health services (20%), with many recruiting from several sources.

The activities to involve stakeholders included the following: focus groups (used for twenty-one technologies), workshops (ten technologies), interviews (nine technologies) and surveys/questionnaires (eleven technologies). Twenty-three technologies (77%) were developed using a triangulation process (i.e. combination of several methods). In addition, specific concepts described include ‘design charrettes’, ‘design jams’ (Hodson et al., 2019), ‘design studios’ (Huggett et al., 2017), ‘crowdsourcing’ and ‘hacka-thons’ (Wiljer et al., 2017), all of which are face-to-face or virtual sessions/spaces to share and develop ideas, and which involve large (e.g. ‘charettes’) or small (e.g. ‘jams’) groups (Tables 1 and 2).

Three articles described using an ‘agile design’ process, which is a dynamic and flexible approach to co-design. This can involve a series of ‘sprint’ cycles that aim to discover, design, develop and test the product, through ‘scrums’ (where one individual leads and another facilitates a team, with predetermined time-frames) or ‘kanbans’ (where team members have specific tasks without fixed-length ‘sprints’; Thabrew et al., 2018).

The SPARX team used the agile design process in the development of the Quest-Te Whitianga CBT app for anxiety and depression. Its development was informed by that of SPARX, starting with the overall learning goals, updated best practice, and a wide scoping consultation exercise (Fleming et al., 2019). A target user group was defined (younger adolescents with a focus on Māori and Pacific YP), and a rapid iteration process was then used, based on ‘sprints’ and ‘scrums’. Three groups were involved: YP, software developers and the research team (including Māori and Pacific researchers). A two-weekly cycle over ten weeks led to the development of five modules, with input from all groups throughout. The app met with initial approval from YP, Māori and Pacific people and clinicians (Christie et al., 2019).

#### Techniques to engage users in activities

In the planning of co-design, studies reported tailoring activities according to the user group, and considering factors such as their age, abilities and health difficulties (e.g. regarding duration, access, media, materials and protocols). For prolonged iterative design, articles described running sessions in spaces that were convenient and appropriate for participants, for example with Quest-Te Whitianga, the workshops were held in the school over lunchtime (Christie et al., 2019). Han et al. (2019) reported difficulties in engaging YP to help guide the development of a suicide prevention app, but successfully organised a Web-based conferencing system, where users did not have to turn on their videos, protecting anonymity. Robinson et al. (2017) found that a closed social media page was a ‘useful and safe’ way to communicate with YP regarding another suicide prevention programme.

The practitioners/researchers participating in the activities, and the expertise and skills required, varied according to the user group, activity, technology and research phase (Table 1). Researchers identified the importance of being comfortable in engaging with CYP and the need to ‘buy-in’ to the collaborative approach (particularly the facilitators), which might be different to controlled research environments. Flexibility, patience and creativity were also needed to guide sessions where users could discuss issues openly (with respect towards others) and allow opportunities for new and interesting ideas to be explored, whilst ensuring that activities were productive and covered relevant user needs and preferences within the time allowed (e.g. Han et al., 2019; Radovic et al., 2018; Thabrew et al., 2018).

Safety and well-being considerations described in the articles include offering refreshments, creating enough space, regular breaks and giving clear ‘ground rules’. To open sessions, there were specific ‘ice breakers’, such as familiar games, and in certain cases the provision of psychoeducation or education on skills relevant to the technology development (Robinson et al., 2017). Interactive exercises included drawing, writing, storytelling, playing, storyboarding, creating videos/animations or virtual/ physical products, and ‘wall storms’ (sticky notes on walls, processed as a group; Fleming et al., 2019). Creative and communication tools included mood-boards, maps, screens and mobile devices to interact with existing technologies and prototypes. Articles outlined safety plans so that help was available in case CYP became distressed or reported difficulties (e.g. suicidal ideation) during the process (e.g. Han et al., 2019). Hodson et al. (2019) ensured that practitioners/facilitators were trained in mental health first aid.

Parents/carers were included in the development of eleven of the technologies (37%). In the scoping phase of Quest-Te Whitianga, evening sessions were held with parents and YP, so that relationships were built with the whole family. This is particularly important in some cultures, for example for Māori and Pacific YP, and allows for consideration about the context for the final delivery of the intervention. There was also a formal opening and closing of sessions by an elder involving a prayer, speech and introductions (Fleming et al., 2019).

### Co-designing the prototype

#### Mapping the prototype

The creation and testing of the initial prototype of the technology was described as an integral part of the co-design process that helped users to engage more with the process. To inform the prototype development, data collection and analysis in the initial design phase were mainly qualitative in nature, based on data from questionnaires, transcripts, notes, images and other materials. When testing the prototypes, Web-usage and computer-screen tracker data were also analysed. ‘Think-Alouds’, where CYP were observed and interviewed whilst using the technologies, were reported as helpful as CYP were ‘immersed’ and ‘less self-conscious’ (Christie et al., 2019; Radovic et al., 2018).

Studies described how the specifications for the content and design of the technology were refined according to the level of importance given to them by the participants and the potential effect on the acceptability, feasibility and ease of use. Other considerations included the programme aims, theory/evidence, technical difficulty, time and resources required, and development costs (Christie et al., 2019; Lucassen et al., 2013; Monshat et al., 2012; Radovic et al., 2018). Whilst twenty-three of the technologies identified (77%) were new or ‘de novo’, the others were adaptations via secondary codesign processes (three from existing digital interventions and four from face-to-face/manualised approaches).

**Figure 2:**
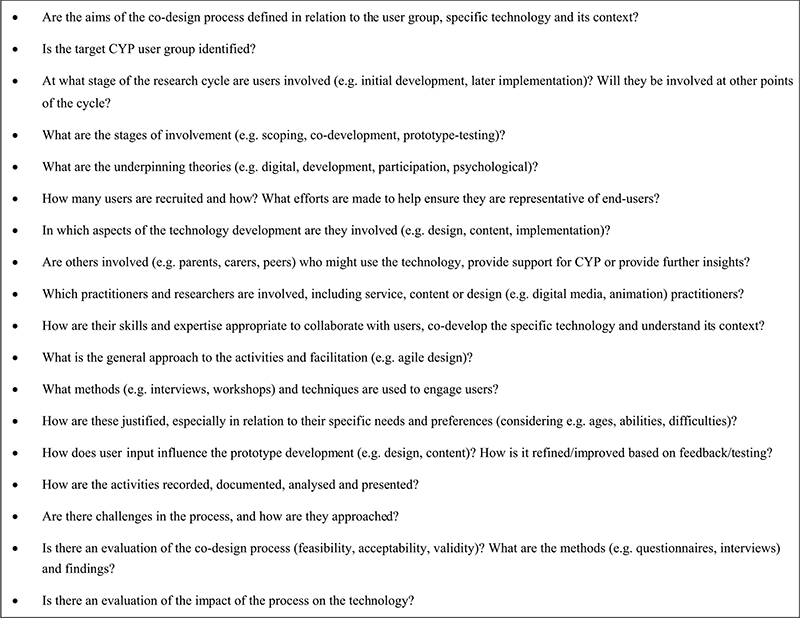
Development of welcome screen and user-flow of MoodHwb: notes/sketches (above), wireframes (centre), early designs (below) (adapted from Bevan Jones, Thapar, Rice, et al., 2018)

Figure 2 shows the development process of the MoodHwb prototype, which was created ‘de novo’. Initial note boards and sketches were created based on user and project requirements, and initial designs were developed in the FGs. Wireframes (skeletal frameworks) were then constructed showing the layout and functionality of each proposed screen, which evolved into the prototype. Discussions with the YP, designers and animator also focused on the illustrations, characters, scripts and animations (Bevan Jones, Thapar, Rice, et al., 2018).

Key frameworks recommend mapping the underlying intervention theory, which can be done in collaboration with CYP, for example through ‘logic models’. This ‘blueprint’ can be referenced and refined throughout the research cycle (Rehfuess et al., 2018). The model can show the possible user activities (e.g. completing exercises, personalising content, gaming), mechanisms of change (e.g. improved understanding, learning self-management skills), potential outcomes (e.g. improvement in symptoms/well-being) and context, including barriers and facilitators to use (e.g. availability of devices, ease of use) when considering implementation (Bevan Jones, Thapar, Rice, et al., 2018).

Potential negative effects were a particular concern in studies. Eighteen articles (72%) highlighted security and confidentiality as important considerations, in part so that CYP engaged with and trusted the technology. Elements to ensure compliance with data protection regulations included the use of usernames and passwords to log-in, locks, moderation, data encryption and secure servers. Choosing a benign brand or name, possibly unrelated to mental health difficulties, can help with privacy (Werner-Seidler et al., 2017).

#### Engaging with the diversity in the group

A range of digital elements were used in technologies to present content and to ensure they had options, increased agency and flexibility (Table 1). Twelve of the technologies used gamification (40%), twelve used interactive exercises/modules (40%), eight included videos/animations (27%), three incorporated social media/messaging (10%) and two used chatbots (7%). The majority were based at least in part on CBT (twenty-two technologies, 73%), whilst seventeen (57%) were based on several psychological or other theories.

Certain studies discussed creating technologies that could be personalised or could address diversity in the user group, for example regarding age/development, gender, cultural context and severity of difficulties (e.g. Lucassen et al., 2013; Werner-Seidler et al., 2017). In a scoping study, Fleming et al., (2019) concluded that younger adolescents who experienced stress or low mood were more likely to be interested in interactive and gamified digital interventions, whilst older adolescents with difficulties were more interested in clearly designed and ‘straight to the point’ products.

Technologies were developed or adapted to engage with specific cultures and subgroups (Saulsberry et al., 2013; Sobowale et al., 2013). Co-design considerations in this context included the language/ text, iconography/symbols, metaphors, colours, characters and, in some cases, the general principles or philosophy of the technology. For example, as noted earlier, the Māori models of mental health are particularly holistic, and this approach influenced the development of SPARX (Shepherd etal., 2015). SPARX has also been adapted for use in Japan, Nunavut and the Netherlands, and to help sexual minority youth (Lucassen et al., 2013).

### Challenges of co-design

During the planning stage, studies described how the flexibility required in the process can lead to difficulties when navigating funding panels and ethics committees that might expect clear plans for the technology. There can be concern about the pace, cost and scale of the process, from users or services. It might be difficult to find the required funds and resources required for co-design activities (e.g. expenses, venues, materials, recordings, transcriptions) and technology development, from research, clinical or other funders. Therefore, authors recommend being clear from the outset about the justification and timescale for this rigorous approach (Hodson et al., 2019; Thabrew et al., 2018).

Some of the challenges related to the methods (e.g. recruitment and engagement) are noted earlier and in Table 2. Another potential risk is that the size or nature of the sample of CYP mean that they are not representative of the target population. In addition, it is likely there is self-selection, in that participants who volunteer are more likely to have an interest in mental health research, although they may still be representative of proposed end-users (Han et al., 2019; Monshat et al., 2012; Radovic et al., 2018; Werner-Seidler et al., 2017). It can be difficult to please all participants when developing the technology, and an attempt to do this can lead to a hybrid that is unacceptable to everyone. To help with these challenges, studies reported efforts to engage a diverse sample where appropriate, and to be clear regarding the need to balance feedback with other considerations (Bevan Jones, Thapar, Rice, et al., 2018; Thabrew et al., 2018). There might also be a difference between what CYP say they want, and what they actually use. A comparison between the input during development and the testing or acceptability stage was described as informative in this regard (Werner-Seidler et al., 2017).

Finally, key guidelines for intervention development recommend assessing the acceptability, feasibility and validity of co-design with CYP (Wight et al., 2016). However, only five articles (19%) described a process evaluation, and none described the impact of the process on the technology. The mixed-methods acceptability evaluations found that participants affirmed the value of collaboration, described the experience as ‘enjoyable’ and ‘rewarding’, stated they had gained knowledge and skills, and felt more able to talk about mental health issues and support others (e.g. Robinson et al., 2017). Negative comments included how activities were ‘exhausting’ (Hodson et al., 2019) and ‘dry’ (Huggett et al., 2017). Wiljer et al. (2017) noted how it had been a challenge to keep YP engaged as the project progressed and they had less ‘ownership’ if they did not have clearly defined roles.

### Practice points and future developments

**Figure 3:**
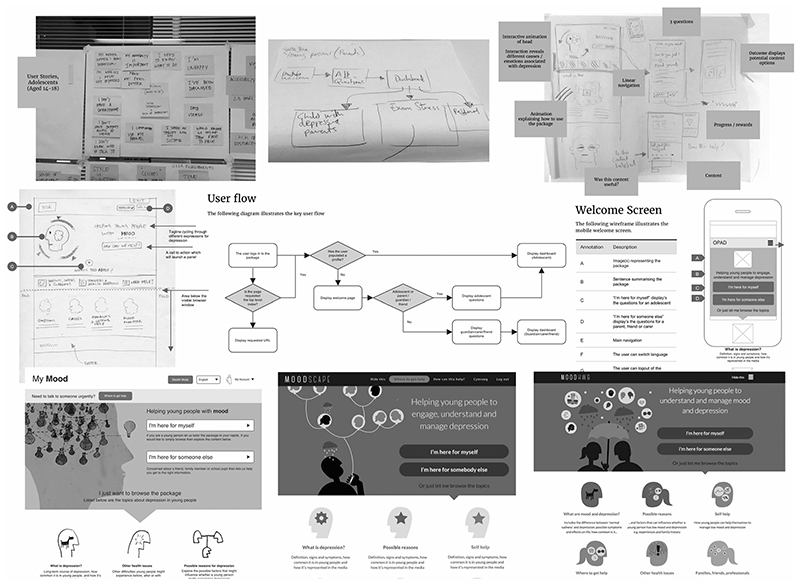
Checklist of questions to consider when planning, reporting or analysing co-design activities

Based on the findings of the review, in Figure 3 we present a checklist of questions that could be considered when planning, documenting or analysing co-design activities. We recommend that practitioners and researchers consider the specific target user group, the technology to be developed and its context (if known), and then align the requirements of the co-design activities accordingly. These issues could be clarified through initial scoping activities.

Furthermore, researchers could consider the needs and preferences of the user group and the heterogeneity within this group, as well as the methods and resources required to recruit and engage them in a collaborative manner on all aspects of the technology, potentially at any point in the research cycle. The practitioners and researchers involved in the process need the necessary skills and expertise (e.g. related to the content, service, design or digital work), to co-develop an engaging, acceptable and helpful technology with and for CYP. A mixed-methods evaluation of the acceptability and feasibility of the process, as well as the potential impact of the process on the technology could be considered.

Given the fast pace of digital technology and culture, there are a number of possible future developments in co-design practice and research. Periodsofreviewbuilt into the research cycle are recommended to future-proof the design and content (Craig et al., 2008). Methods of co-design might adapt as technologies become more complex, flexible and personalised. There might be more ‘virtual’ groups and workshops, which can help with reach and access a more diverse group of users (Han et al., 2019). Evaluations of the impact of co-design might involve trials comparing codesigned technologies with those that did not involve CYP or used alternative models.

There are concerns about the timeframe of the pipeline from development to implementation, particularly with the need for faster translation of findings into the community. There is also the challenge of validating a moving target that becomes irrelevant if pausing for long. More flexible and quicker models in the ‘real world’ will be needed, for example using digital ecosystems with built-in architecture to support rapid re-testing of different versions using a range of measures (Rice et al., 2018; Thabrew et al., 2018), but maintaining a rigorous approach to development and evaluation, with no harm and minimal costs. All these developments might involve collaboration between academic, clinical and commercial sectors, and there might be learnings from gaming and commercial apps (Christie et al., 2019).

## Discussion

We have conducted a review of the use of co-design in the development of digital mental health technologies with CYP and have supplemented this with case studies and practice points based on the findings and from several researchers with experience of co-design practice with this age group. There is a range of approaches to involve CYP and other stakeholders in the co-development of technologies throughout the research cycle. These methods need to be tailored according to the users (considering the diversity within the group), digital technology and setting. There are also potential challenges, in particular related to finding the resources required, balancing the input of all stakeholders and evaluating the impact on technologies. The review helps to inform practitioners and researchers interested in developing technologies for CYP.

The increase in articles published over recent years on the design and development of technologies with CYP, suggests there is increased interest in this field - especially since the publication of a systematic review by Orlowski et al (2015) on YP involvement in the design of technology-based interventions (although this focused on ‘youth’ and had a broader approach to participation, mental health/well-being and interventions). The earlier review concluded that YP involvement was mainly consultative in nature, whilst our review suggests that such activities might have become more collaborative. The lack of documented evaluations in our review is consistent with Orlowski et al’s findings that there was limited outcome data and evidence on the impact of participatory research on intervention effectiveness.

The strengths of our review include the systematic approach to the search and the collation of information by practitioners and researchers in this field from around the world. This is the first review, to our knowledge, that brings together co-design practices of digital mental health technologies for children as well as YP, although it builds on the previous review.

The review has limitations. We acknowledge that other technologies (e.g. for depression or anxiety) might have involved CYP, but were not captured by this search (e.g. because this was not documented clearly in peer-reviewed papers). Whilst the review focused on resources/interventions for specific mental health difficulties, co-design also plays an important role in technologies used in other areas of mental health, as well as for physical health, and in assessment, communication and data management. There are also other models of involving stakeholders, as well as participatory design and co-design (Orlowski et al., 2015), although we took an inclusive approach to screening articles regarding CYP involvement. We did not appraise the quality or effectiveness of activities, given the heterogeneity of practices and lack of guidance in this field. However, this could be a focus of future papers as the field develops. The practitioner points section includes some of the authors’ views, and there are likely to be other perspectives not represented here.

Whilst there are emerging studies involving YP and certain subgroups in co-design, younger children and those with learning disabilities and specific difficulties are under-represented and will have specific needs and preferences. More research is also required into the implementation phase of technologies, and how co-design can play a part (Craig et al., 2008). Most of the studies identified were based in ‘developed’ countries, and there is increasing interest in the use of digital technologies in LMIC (Naslund et al., 2017). If the field is to progress and have genuine lasting impact, further research and guidance are required on processes to involve CYP and their evaluation. The co-design practices might then become the new benchmark for how digital technologies of high quality are developed.

## Supplementary Material

Figure S1

## Figures and Tables

**Table 1:** Digital mental health technologies for CYP and their development approaches

Digital programme (Authors, countiy)	MH difficulties	Stakeholder involvement (ages of CYP in years, if stated)	Technological approaches	Psychological & other theories
Adventures of DoReMiFa (Shum et al., 2019; Hong Kong)	Anxiety, general MH	FGs - children (8-12), parents, teachers, practitioners	Gamification, storytelling	CBT, positive psychology
LifeBuoy (Han et al., 2019; AWS-author^a^; Australia)	Suicidal thoughts	Surveys/FGs - YP (16-25)	Interactive modules, gamification	DBT, ACT
Mellow (Hodson et al., 2019; Canada)	General MH, crises	Interviews/workshops/surveys (‘design charrettes/jams’ - YP (13-24), friends, families/carers, practitioners	Joumal/planning companion tool	Holistic crisis planning
Quest-Te Whitianga (Christie et al., 2019; Fleming et al., 2019; SM, KS-authors^a^; New Zealand)	Anxiety, depression	Interviews/FGs/workshops (‘wall storms’/ Think-Alouds’ - YP (12-25), designers, practitioners	Modular activities, gamification	CBT, positive psychology, mindfulness, interpersonal skills
Bluelce (Stallard et al., 2018; PS-author^a^; England)	Self-harm	Meetings/workshops - YP (12-17), practitioners, developers	Mood diary, mood-lifting activities, safety checks	CBT, DBT
HABITs (Thabrew et al., 2018; SM-author^a^; New Zealand)	Emotional health, substance use	Surveys/FGs - YP, practitioners, cultural advisors	Different user groups: games, chatbots, intrinsic motivators; digital eco-system	CBT, positive psychology, harm minimisation
MoodHwb (Bevan Jones, Thapar, Rice, et al., 2018; RBJ, FR, SSA, PS, SM, SAS-authors^a^; Wales)	Depression	Interviews/workshop/FGs - YP (13-19), parents/carers, practitioners, designers	Illustrations / animations, profile-builder, moodmonitor, goal-setting	Psychoeducation, CBT, social support
Rebound (Rice et al., 2018; SR, MAJ-authors^a^; Australia)	Depression	Workshops/FGs/consultations - YP (15-25), families, professionals, writers/artists, designers	Social media-enabled platform	CBT, mindfulness, positive psychology, social support
SOVA (Radovic et al., 2018; USA)	Depression, anxiety	Interviews (‘Think-Alouds’/FGs - YP (13-26), parents, advocates, professionals	Moderated social media	Social support, psychoeducation
BeSafe (Huggett et al., 2017; Canada)	General MH, addictions, crises	‘Design studio’/meetings - YP, practitioners	Navigation, safety plans, decision aid	Empowerment, social support
Sleep Ninja (Werner-Seidler et al., 2017; AWS-author^a^; Australia)	Sleep, depression	Interviews/FGs/consultations - YP (12-16), parents, professionals, designers	Chatbot, gamification	CBT-I
Social media messages (Robinson et al., 2017; Australia)	Suicidal thoughts	Closed social media/surveys/workshops - YP (16-18), creative agency	Social media messages/videos	Psychoeducation, social support
Thought Spot (Wiljer et al., 2017; Canada)	General MH	‘Crowdsourcing’/ ‘hackathon’/ workshops, FGs - YP (15-24), practitioners, designers	Information sharing, networking	Peer/social support
SPARX (Shepherd et al., 2015; SM, KS-authors^a^; New Zealand)	Depression	Workshops/FGs - YP (13-18), families, clinicians, designers, cultural advisors	Gamification, avatars	CBT
CLIMATE Schools (Teesson et al., 2014; Australia)	Depression, anxiety, substance misuse	FGs - YP (13-15), practitioners, designers	Interactive modules, illustrated storylines	Psychoeducation, CBT, harm minimisation
CURB (Saulsberry et al., 2013; USA)	Depression	Surveys/workshops - YP (15-18), parents, practitioner	Interactive modules	CBT, IPT
Grasp the opportunity (Sobowale et al., 2013; Hong Kong)	Depression	Questionnaires/FGs/discussions - YP, parents, teachers, practitioners	Interactive modules	CBT
Rainbow SPARX (Lucassen et al., 2013; SM, KS-authors^a^; Australia)	Depression	Questionnaires/FGs - YP (16-27)	Gamification, avatars	CBT
Digital programme (Authors, country)	MH difficulties	Stakeholder involvement (ages of CYP in years, if stated)	Technological approaches	Psychological & other theories
MATE (Monshat et al., 2012; Australia)	General MH	Interviews - YP (16-26)	Interactive modules, videos, forum	Mindfulness
MEMO (Whittaker et al., 2012; SM, KS-authors^a^; New Zealand)	Depression	FGs - YP (13-17), practitioners, mHealth experts	Text messages, videos/ animations	CBT
Stressbusters (Robinson et al., 2011; P. Abeles^a^, 26.2.20; England)	Depression	Surveys/FGs - YP, practitioners, designers	Interactive sessions, videos	CBT
Think-Feel-Do (Stallard et al., 2011; PS-author^a^; England)	Depression, anxiety	FGs - CYP (11-16), designers	Interactive sessions, videos	CBT
CATCH-IT (Landback et al., 2009; USA)	Depression	Groups/questionnaires - YP, practitioners	Interactive modules	CBT, IPT
Reach Out! (Oliver et al., 2006; Australia)	General MH	Forums/mixed-methods - YP (16-25), practitioners	Forum, gaming, podcasts, blogs	CBT, social support
Technologies identified via personal communication (articles describing CYP involvement not available) BRAVE-ONLINE (S.March^a^, 21.2.20; Australia)	Anxiety	Surveys/FGs - CYP (7-18), practitioners	Interactive sessions, animations, games	CBT
Mightier (JK-author^a^; USA)	Emotional regulation	Observations/FGs/Interviews - CYP (6-14), parents / carers	Games, biofeedback	Mindfulness, constructivism
MoodGYM (H.Christensen^a^, 20.2.20; Australia)	Depression, anxiety	Interviews/FGs - YP	Interactive modules, workbook	CBT
Pesky/Mindful gNATs (G.O’Reilly^a^, 24.2.20; Ireland)	Anxiety, depression	FGs - CYP (9-17), practitioners, designers	Gamification	CBT, mindfulness
Smooth Sailing (M.Subotic-Kerry^a^, 30.9.19; Australia)	Depression, anxiety	Surveys/interviews, FGs - YP (13-16), counsellors, GPs, parents	Stepped-care, interactive modules	Psychoeducation, CBT, counsellor referral
The Journey (KS, SM-authors^a^; New Zealand)	Depression	Workshops - YP, designers	Gamification, videos/ animations	CBT

DBT, Dialectical behaviour therapy; IPT, Interpersonal psychotherapy; MH, Mental health.

a Personal communication.

**Table 2:** Examples of methods of involvement

Description	Potential benefits	Potential challenges	Approaches to engage participants
Questionnaires/surveys (Paper, digital)	Large amount of data, range of participants, increased reach, accessibility, economical, less intrusive	Difficult to explore issues in-depth, poor engagement	Engaging documents; digital: progress-bars, multiplatform approach, videos/animations
Interviews (Face-to-face, telephone/digital)	Explore in-depth & new issues, participants can define agenda & choose setting, interaction with prototype, high credibility & face validity	Intrusive, timeconsuming, reluctance to give critical feedback	‘Think-Alouds’ (participant observed/interviewed whilst using technology)
Focus groups (Face-to-face, digital/‘virtual’	Explore breadth of issues & new ideas, involve diverse group of CYP & other stakeholders, interaction with prototype, more economic/ efficient than interviews	Difficult to talk to ‘strangers’ in new setting, social biases (e.g. conformity), travel to face-to-face groups	Ground rules, screens & devices, materials
Interactive workshops/meetings (Face-to-face, digital/‘virtual’	As with FGs; less formal, range of interactive activities	As with FGs; difficulties with recording, transcription & analysis	As with FGs; ‘Wall storms’ (sticky notes on walls, processed as a group), ‘Word clouds’ (words used commonly/prominently grouped together)
‘Design studios’ (intensive development sessions) (Huggett et al., 2017)			
‘Design charrettes’ (larger meetings e.g. to sketch/storyboard ideas)			
‘Design jams’ (smaller sessions e.g. to develop multiple iterations of user experiences) (Hodson et al., 2019)			
‘Crowdsourcing’ (open call to large group, often online e.g. to contribute project content)			
‘Hackathon’ (digital event with large group e.g. proposing ideas for technologies) (Wiljer et al., 2017)			
Observations/ethnographic approaches	Understanding context & implementation, identify unexpected issues, detailed/‘faithful’ representation of behaviours & preferences	Time-consuming, CYP may not act ‘naturally’	Appreciation & respect for environment

## Key points

- The aim of co-design is to ensure that digital technologies meet the users’ needs and preference, so that the technologies are potentially more engaging, feasible, acceptable and effective.
- Co-design can feature throughout the life cycle of the technology and in all stages of the research including development, evaluation and implementation.
- There is a range of methods to involve and engage children, young people and other stakeholders in codesign.
- Future work in this field will need to consider the changing face of technology, the diversity of the user group, the need for faster translation of findings into the community, and the evaluation of the co-design process and its impact.
