# Supplementary material for: Practitioner review: Co-design of digital mental health technologies with children and young people
Source: J Child Psychol Psychiatry. Author manuscript; Available in PMC 2021 Nov 10. (PMC7611975; doi:10.1111/jcpp.13258)
Supplement: Figure S1 [file EMS137683-supplement-Figure_S1.docx]

**Supporting information – Practitioner review: Co-design of digital mental health technologies with children and young people – by Bevan Jones *et al*.**

**Figure S1.** Flow diagram – methodology for article selection.

**Search terms, inclusion criteria**

teen* or young or youth or adolescen* or child* or paediatric or pediatric,

and mental health or mental or psycholog* or psychiatr*, and internet or social media or telemedicine or cellular phone or mobile phone or smartphone app* or mobile app* or digital or web-based or website* or e-health or m-health or online or computer*, and design* or develop* or produc* or codesign* or codevelop* or coproduc*

Focus on depression, anxiety, sleep, self-harm, suicide

Digital mental health technologies (resources and interventions)

to help CYP (up to 18 years old)

English/translated into English

No restriction regarding publishing dates

**Database searches**

Ovid used for searches in July 2019, Mendeley software used to manage data;

RBJ screened all articles, SSA screened 20% of articles

– both extracted data from articles included in review

Medline PsycINFO Web of Science

n=3283 n=2030 n=3651

Reasons for exclusion:

No clear description of involvement of CYP in the design/development

Technologies developed for:

adults

other mental health difficulties

physical health

Diagnostic, screening, monitoring, communication or data management tools

Articles screened from title/abstracts

(after duplicates removed)

n=5891

Articles assessed from full-text

n=292

Full-text articles identified through: reviews, guidelines,

reference lists,

authors’ personal collections, contacting key authors

n=7

Articles included in review

n=25

(describing 24 digital technologies*)

**Six additional technologies were identified via personal communication. However, articles describing CYP involvement in their design/development were not available.*
